# Facile Synthesis of Flexible Methylsilsesquioxane Aerogels with Surface Modifications for Sound- Absorbance, Fast Dye Adsorption and Oil/Water Separation

**DOI:** 10.3390/molecules23040945

**Published:** 2018-04-18

**Authors:** Xingzhong Guo, Jiaqi Shan, Zhongzhang Lai, Wei Lei, Ronghua Ding, Yun Zhang, Hui Yang

**Affiliations:** 1 School of Materials Science and Engineering, Zhejiang University, Hangzhou 310027, China; 21626008@zju.edu.cn (J.S.); 3150102792@zju.edu.cn (Z.L.); yanghui@zju.edu.cn (H.Y.); 2 Pan Asia Microvent Tech (Jiangsu) Coporation & Zhejiang University Micro-nano-porous Materials Joint Research Development Center, Changzhou 213100, China; leiwei@microwent.com.cn (W.L.); dingronghua@microvent.com.cn (R.D.); zhangyun@microvent.com.cn (Y.Z.)

**Keywords:** MSQ aerogel, flexibility, sol-gel, surface modification, sound-absorbing, dye adsorption, oil/water separation

## Abstract

New flexible methylsilsesquioxane (MSQ) aerogels have been facilely prepared by a sol–gel process with methyltrimethoxysilane (MTMS) and dimethyldimethoxysilane (DMDMS) as co-precursors, followed by surface modification and ambient pressure drying. The microstructure, mechanical properties and hydrophobicity of these MSQ aerogels after surface modifications of hexamethyldisiloxane (HMDSO) and/or hexamethyldisilazane (HMDS) were investigated in detail, and the applications of surface-modified MSQ aerogels in sound-absorbance, fast dye adsorption and oil/water separation were evaluated, respectively. The MSQ aerogels surface-modified by HMDS possess flexibility, elasticity and superhydrophobicity, and demonstrate good performance in the mentioned applications. The resultant MSQ aerogel used in sound-absorbance has high frequency (about 6 kHz) acoustic absorptivity of up to 80%, benefiting from its macroporous structure and porosity of 94%, and it also possesses intermediate frequency acoustic absorptivity (about 1 kHz) up to 80% owing to its elasticity. This MSQ aerogel can selectively separate oil from oil/water mixtures with high efficiency due to its superhydrophobicity and superlipophilicity, resulting from a lot of methyl groups, density as low as 0.12 cm^3^·g^−1^ and a water contact angle as high as 157°. This MSQ aerogel can be assembled to be a monolithic column applied for fast dye adsorption, and shows selective adsorption for anionic dyes and removal efficiency of methyl orange of up to 95%.

## 1. Introduction

Methylsilsesquioxane (MSQ) aerogel [1,2] is a kind of methyl hybrid SiO_2_ aerogel monolith material, derived from organoalkoxysilanes with methyl groups through a facile sol-gel process [3,4,5]. The sol-gel process is a wet chemical preparation method in chemistry and material science field [6,7], because sol-gel derived materials usually possess many advantages, such as high purity, well-distributed chemical composition and adjustable microstructure. Because of the introduction of methyl groups, the structures of the MSQ aerogels will be different from the conventional three-dimensional space network structures of traditional silica aerogels, which tend to be non-contiguous networks of bead chains [8]. Meanwhile, benefitting from the introduction of methyl groups, MSQ aerogels possess better flexibility, hydrophobicity and lipophilicity than traditional silica aerogels, which can be applied for sound-absorbance, dye adsorption, oil/water separation, shock resistance, lossless liquid transmission applications, and so on [8,9].

In recent years, many studies have been devoted to the synthesis of MSQ aerogels, especially using various silane coupling agents as precursor. Methyltrimethoxysilane (MTMS) is a common precursor for MSQ aerogels. Rao et al. [10,11] firstly reported the preparation of flexible MSQ aerogels using MTMS as precursor, oxalic acid as catalyst, methanol as solvent and ammonia as gelation agent via supercritical drying (SCD), while it needs special conditions, such as high pressure and high temperature, precluding the extended production and application of these aerogels. Therefore, on the basis of Rao’s system, Liang Zhong et al. [12] used polydimethylsiloxane (PDMS) and methyltriethoxysilane (MTES) as co-precursors to successfully prepare flexible MSQ aerogels with elastic limit of 60% and contact angle of 150.8° via atmospheric pressure drying. However, the introduction of PMDS (high polymer) brings about clustering of methyl groups leading to structure instability and inhomogeneity of the resultant MSQ aerogels.

In this study, new flexible methylsilsesquioxane (MSQ) aerogels have been easily prepared by a sol–gel process using methyltrimethoxysilane (MTMS) and dimethyldimethoxysilane (DMDMS) as co-precursors, dilute hydrochloric acid as catalyst and solvent, propylene oxide (PO) as gelation agent and hexadecyltrimethylammonium chloride (CTAC) as surfactant with surface modifications of hexamethyldisiloxane (HMDSO) and/or hexamethyldisilazane (HMDS), followed by ambient pressure drying. The surface modifications were found to have a remarkable effect on improving the flexibility and hydrophobicity of MSQ aerogels, and the surface-modified MSQ aerogels demonstrate superior performances in the fields of sound-absorbance, fast dye adsorption and oil/water separation, respectively.

## 2. Results and Discussion

### 2.1. Synthesis of New Flexible MSQ Aerogels

**Figure 1:**
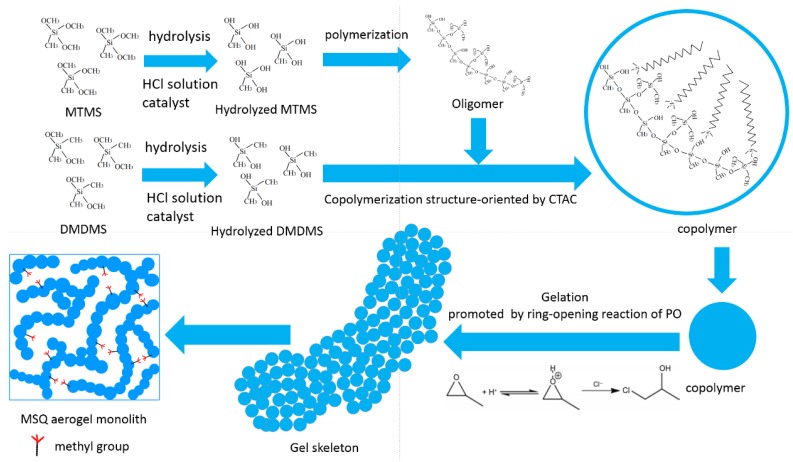
Reaction scheme of the sol–gel process for the MTMS–DMDMS derived MSQ aerogel.

Figure 1 shows the reaction scheme of the sol–gel process for the MTMS–DMDMS derived MSQ aerogel. Catalyzed by HCl solution, MTMS hydrolyzes and polymerizes to form oligomers. With the addition of DMDMS, copolymerization structure-oriented by CTAC between oligomer and hydrolyzed DMDMS occurs to form a copolymer, which is determined by the addition order of co-precursors. The copolymer further polymerizes to form gel skeletons, which are gelated by means of the ring-opening reactions of PO units.

**Figure 2:**
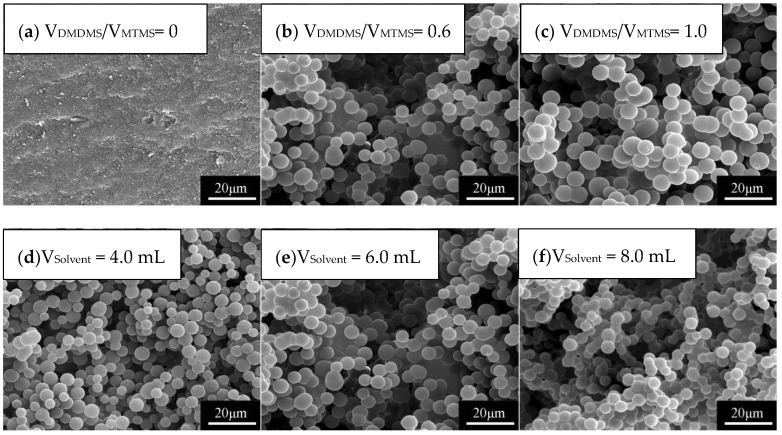
SEM images of flexible MSQ aerogels (without surface modification) prepared with varied volume ratios of co-precursors and solvent volumes (total volume of co-precursors is 2.0 mL).

The microstructure of the aerogel frameworks are significantly affected by co-precursor volume ratio and solvent volume. Figure 2a–c show SEM images of flexible MSQ aerogels prepared with varied volume ratios of co-precursors. With the addition of DMDMS, the microstructure of aerogels changes from dense to a non-contiguous network of bead chains. The volume ratio of co-precursors determines the amount of methyl groups in the sol-gel system, which in turn influences the phase separation during the gelation. More DMDMS will increase the amount of methyl groups, which will improve the phase separation of the system. When little phase separation occurs, the microstructure of the MSQ aerogel is dense without pores (Figure 2a). With the development of phase separation, the microstructure of MSQ aerogel becomes a network of bead chains (Figure 2b). When too much phase separation occurs, only precipitates (i.e., no gelation) can be seen. Figure 2d–f show SEM images of flexible MSQ aerogels prepared with varied solvent volumes. With the increase of the volume of solvent, the pore diameter of the aerogels becomes larger. According to the theory of sol-gel process accompanied by phase separation [4,5], the pores of aerogels are derived from the removal of solvent. This indicates that the pore diameter is determined by the amount of solvent, and more solvent will lead to a bigger pore diameter.

**Figure 3:**
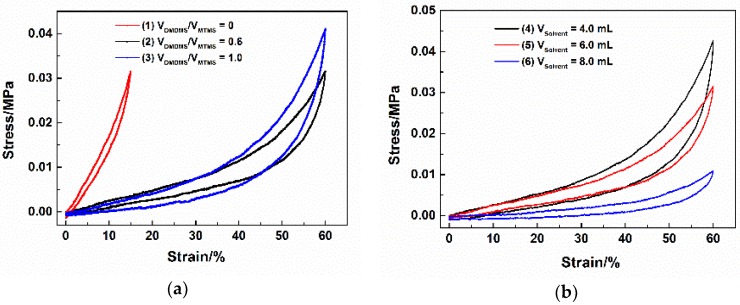
(**a**) Stress-strain curves of flexible MSQ aerogels (without surface modification) prepared with varied volume ratios of co-precursor; (**b**) Stress-strain curves of flexible MSQ aerogels (without surface modification) prepared with varied solvent volumes.

The elasticity of flexible MSQ aerogels was evaluated by uniaxial compression tests, in which all MSQ aerogels could be compressed and sprang back. Figure 3a shows the stress-strain curves of flexible MSQ aerogels prepared with varied volume ratios of co-precursors. With the addition of DMDMS, the elasticity of MSQ aerogels is significantly enhanced (the elastic limit increases from 10% to 60%, while the Young’s modulus declines from 1.94 to 0.25 KPa), which benefits from the increase of methyl groups. With the increase of the volume ratio of DMDMS/MTMS, the amount of methyl groups in the MSQ aerogel increases while the amount of Si-O decreases, as the interaction force between methyl groups results in MSQ aerogel elasticity and the amount of Si-O influences the rigidity of the MSQ aerogels. This implies that a bigger volume ratio of DMDMS will bring about better elasticity and the worse rigidity. When the volume ratio of DMDMS/MTMS is 0.6, the MSQ aerogel presents the lowest Young’s modulus (0.26 KPa) and better spring back, indicating the best elasticity. Figure 3a shows the stress-strain curves of flexible MSQ aerogels prepared with varied solvent volumes. It is seen that the elasticity of MSQ aerogels increases with the increase of solvent amount, and also increases after surface modification. As is well known, the higher solvent amount indicates a larger pore diameter and porosity, which makes aerogel easier to compress and more fragile. With the increase of solvent volume, the Young’s modulus of the MSQ aerogels gradually decreases from 0.28 to 0.07 KPa. According to the curves, the aerogel prepared with 8 mL of solvent seems to have the best elasticity. Although increasing solvent volume has a significant effect on the elasticity, it also reduces the toughness, which will limit the applications of the flexible MSQ aerogels.

**Figure 4:**
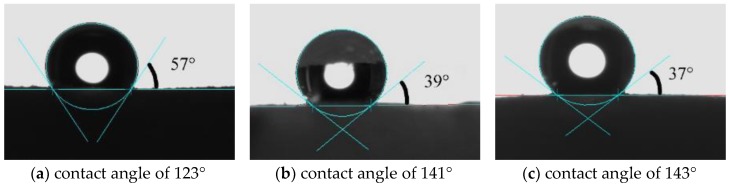
Contact angle images of flexible MSQ aerogels (without surface modification) prepared with varied volume ratios of co-precursor: (**a**) V_DMDMS_/V_MTMS_=0; (**b**) V_DMDMS_/V_MTMS_ = 0.6; (**c**) V_DMDMS_/V_MTMS_ = 1.0.

Hydrophobicity is characterized by the water contact angle of flexible MSQ aerogel. Figure 4 shows the contact angle images of flexible MSQ aerogels (without surface modification) prepared with varied volume ratios of co-precursors. With the addition of DMDMS, the contact angel of aerogels gradually becomes larger, because the addition of DMDMS increases the amount of methyl groups on the surface of aerogel, which has a significant effect on the hydrophobicity of MSQ aerogels.

### 2.2. Surface Modification of Flexible MSQ Aerogels

**Figure 5:**
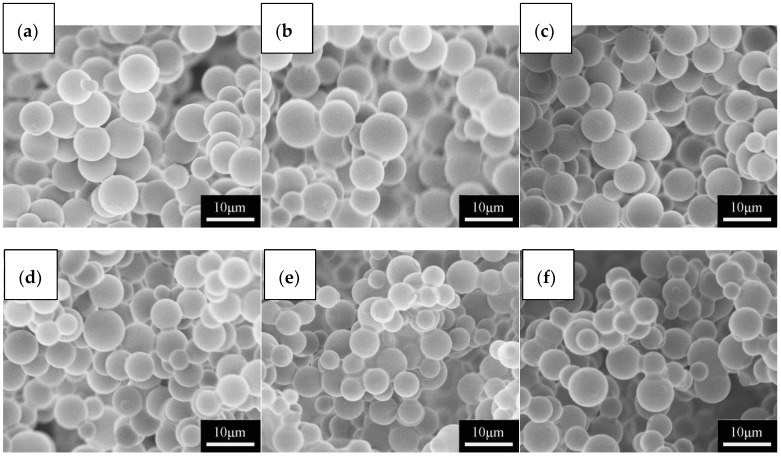
SEM images of flexible MSQ aerogels with different surface modifications: (**a**) HMDSO; (**b**) HMDS; (**c**) HMDSO + HMDS (volume ratio is 1:1); (**d**) HMDSO + IPA (volume ratio is 0.4:10); (**e**) HMDS + IPA (volume ratio is 0.4:10); (**f**) HMDSO + HMDS + IPA (volume ratio is 0.2:0.2:10).

With comprehensive consideration of microstructure, elasticity and hydrophobicity, we chose a flexible MSQ aerogel prepared with 0.6 of co-precursor volume ratio and 6 mL of solvent for further surface modification and application evaluation. Figure 5 shows the SEM images of flexible MSQ aerogels with different surface modifications. After surface modifications, the microstructure of flexible MSQ aerogels remains a non-contiguous network of bead chains. However, compared with Figure 2, the bead chains become thicker, which is explained by a large amount of –Si(CH_3_)_3_ groups grafted on the surface of gel skeletons by surface modification. Equation (1) shows the corresponding reaction [13]:

≡Si-OH + (CH_3_)_3_SiNHSi(CH_3_)_3_ = NH_3_+ ≡Si-O-Si(CH_3_)_3_ (1)

Equation (1) describes the reaction of OH groups and HMDS on the MSQ aerogel, which is similar to HMDSO. It indicates that HMDS has the same effect as HMDSO. IPA is used to adjust the concentration of these two surface modifiers. MSQ aerogels surface-modified with higher concentration of HMDS or HMDSO (Figure 5a–c) present thicker bead chains than those surface-modified with lower concentration of HMDS or HMDSO (Figure 5d–f) because there are more Si(CH_3_)_3_ groups grafted on the surface of gel skeletons with more surface modifiers.

**Figure 6:**
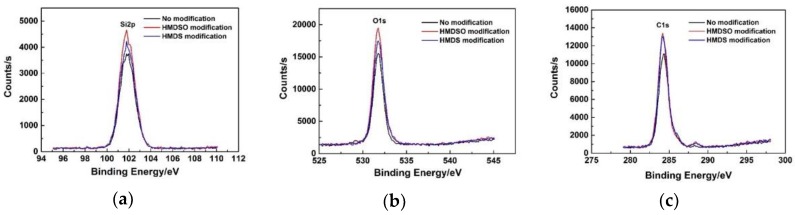
(**a**) Si2p, (**b**) O1sand (**c**) C1s XPS spectra of flexible MSQ aerogels without or with surface modifications.

To characterize the chemical composition of the aerogels and verify the effect of surface modification, the XPS spectra of flexible aerogels were measured. Figure 6 shows the XPS spectra of flexible MSQ aerogels without or with surface modification. From the XPS spectra, the Si, O and C of aerogels without or with surface modifications have the same corresponding XP levels, which proves that their chemical composition (on the surface, detection depth of XPS is about 3~5 nm) can be described as SiO_x_(CH_3_)_y_.

By calculating the area of the fitted spectral peaks, the atomic percentages of flexible MSQ aerogels can be obtained, as shown in Table 1. Substituting the atomic percentage into SiO_x_(CH_3_)_y_, the surface composition of aerogels and the proportion of methyl groups in these formulas are much larger than that of DMDMS, proving that the methyl groups have clustered on the surface of aerogels. Meanwhile, after surface modification by HMDS, there are more methyl groups on the surface of the aerogel, which indicates that methyl groups have been successfully grafted on the surface of the flexible MSQ aerogel.

**Figure 7:**
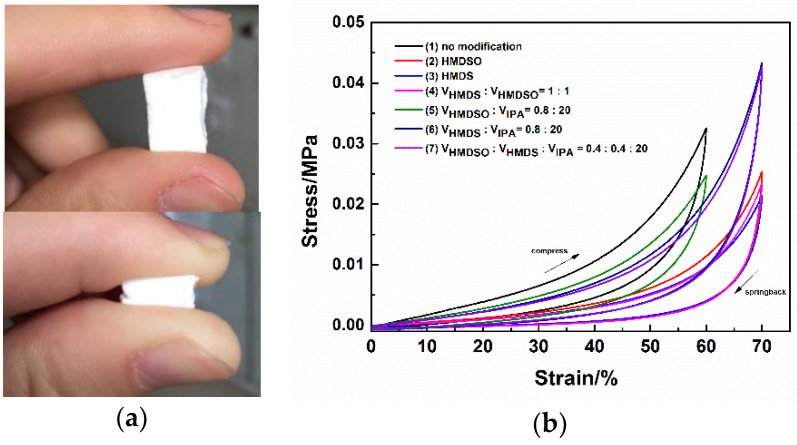
(**a**) Digital camera images of flexible MSQ aerogels before and after stressed; (**b**) Stress-strain curves of flexible MSQ aerogels with different surface modifications.

The elasticity of flexible MSQ aerogels with different surface modifications has also been evaluated by uniaxial compression tests. Figure 7b shows the stress-strain curves of flexible MSQ aerogels with different surface modifications. Maximum strain, maximum stress and Young’s modulus of flexible MSQ aerogels with different surface modifications have been recorded in Table 2 from their stress-strain curves. As shown in Table 2, after surface modifications, the elastic limit of MSQ aerogel increases from 60% to 70% and the maximum stress decreases, indicating that surface modification can effectively enhance the elasticity of MSQ aerogel. MSQ aerogels surface-modified with higher concentrations of HMDS or HMDSO (Samples (1)–(3)) have higher elasticity, which results from more methyl groups grafted on the surface of gel skeletons with more surface modifiers. Since HMDS has higher reactivity than HMDSO, MSQ aerogels with HMDS surface modification display the lowest Young’s modulus, suggesting the highest elasticity.

**Figure 8:**
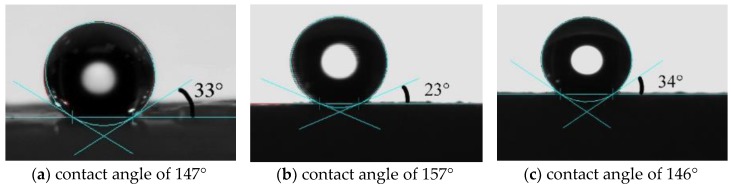
Contact angle images of flexible MSQ aerogels with different surface modifications: (**a**) HMDSO; (**b**) HMDS; (**c**) HMDSO + HMDS (volume ratio is 1:1).

Figure 8 shows the water contact angle images of flexible MSQ aerogels with different surface modifications: (a) HMDSO; (b) HMDS; (c) HMDSO + HMDS (volume ratio is 1:1). According to Figure 4 and Figure 8, the contact angle of aerogels with surface modifications is higher than that of aerogels without surface modification, especially the contact angle of aerogel with modification of HMDS increases to 157°, which corresponds to superhydrophobicity.

### 2.3. Sound-Absorbing Test of New Flexible MSQ Aerogels

**Figure 9:**
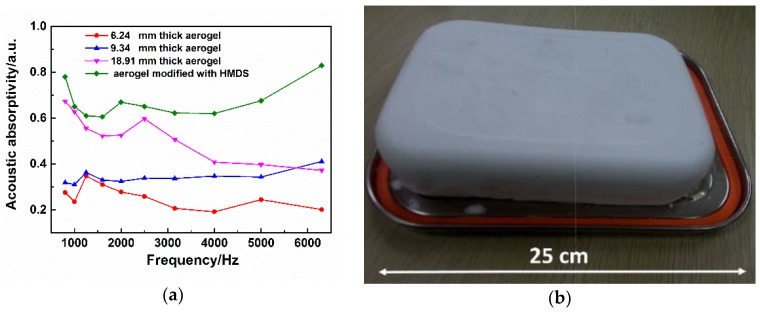
(**a**) Acoustic absorptivity curves of flexible MSQ aerogels measured by standing wave tube method; (**b**) Digital camera image of MSQ aerogel prepared with equal proportions, magnified 200 times.

Since aerogels were first investigated as low sound speed materials, more and more studies about sound the absorption of aerogels were reported, most of which were related to soft organic fiber-composite SiO_2_ aerogels [14,15,16,17,18,19,20], such as silica–cellulose hybrid aerogels, polyurethane/silica aerogels, polyethylene terephthalate fiber nonwovens/silica aerogels, unplastisized polyvinyl chloride/silica aerogels and so on. All of these aerogel composites possess high flexibility and porosity, which inspired us to test the sound absorption of the resultant MSQ aerogels with similar properties. Figure 9b shows an intact MSQ aerogel prepared with equal proportions magnified 200 times. By measuring the volume and mass of the aerogel, its density and porosity are calculated to be 0.12 g·cm^−3^ and 94%, respectively. The acoustic absorptivity of flexible MSQ aerogels was measured by the standing wave tube method. Figure 9a shows the acoustic absorptivity curves of flexible MSQ aerogels. With the increase of thickness of the aerogels, their acoustic absorptivity gradually increases, especially at intermediate frequencies (about 1 kHz). The increase in aerogel thickness heightens the collision frequent between sound waves and gel skeletons, and reduces the energy of reflected waves, resulting in high acoustic absorptivity. It is also found from Figure 9a that the MSQ aerogel with surface modification of HMDS possesses high frequency acoustic absorptivity (about 6 kHz) up to 80%, benefitting from its macroporous structure and high porosity of 94%. Also, it possesses an acoustic absorptivity of intermediate frequency (about 1 kHz) of up to 80% owing to its high elasticity. MSQ aerogels with surface modification of HMDS have higher acoustic absorptivity than MSQ aerogels without surface modification, resulting from the increase of methyl groups on the surface of the gel skeletons. The increase of methyl groups on the surface of gel skeleton is propitious to improve the elasticity of gel skeleton, which is inferred to have a significant effect on energy losses resulting from the collisions between sound waves and gel skeletons. Since the essence of sound waves is vibration, a surface with higher elastic tends to have better damping effects.

### 2.4. Oil/Water Separation Properties of the New Flexible MSQ Aerogels

**Figure 10:**
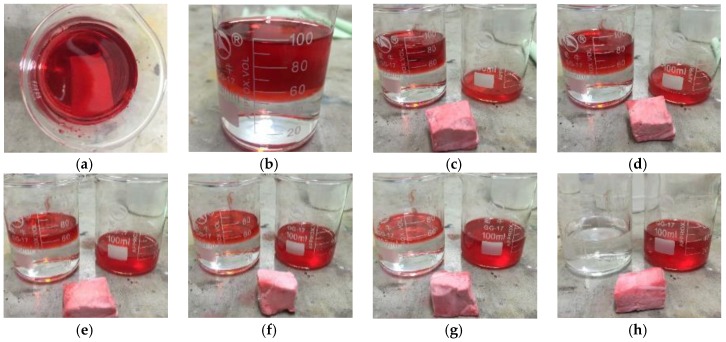
Digital camera images of oil/water separation experiment process; (**a**) aerogel in mixture; (**b**) aerogel in mixture; (**c**) first absorption; (**d**) second absorption; (**e**) third absorption; (**f**) fourth absorption; (**g**) fifth absorption; (**h**) sixth absorption.

To solve the problems of oily waste water and frequent oil spill accidents, researchers have started to pay attention to hydrophobic aerogels with high porosity for their oil absorption capacity and high hydrophobicity for oil/water separation. Over the past few years, various hybrid silica aerogels were reported for efficient oil/water separation [8,21,22,23,24,25,26]. Owing to the great elasticity, high porosity, superhydrophobicity and superlipophilicity of MSQ aerogels with HMDS surface modification, a simple oil/water separation experiment of the resultant surface-modified MSQ aerogels was carried out. Firstly, 100 mL of oil/water mixture with 50 mL of water and 50 mL of *n*-heptane was prepared and marked by Oil Red O. Secondly, a piece of MSQ aerogel (weighing 1.2 g) was put into the oil/water mixture where it floated in the oil phase (Figure 10a,b) due to its low density and superhydrophobicity. Then, the aerogels was taken out by tweezers and the absorbed oil was squeezed out into another beaker. This is repeated six times (Figure 10c–h) and finally all of oil was completely separated from water. Every time about 9.5~10 mL of oil could be squeezed out, and the last time about 1~2 mL of oil was squeezed out. Residual oil in the aerogel was calculated to be about 1.6 g, and the oil absorption capacity of MSQ aerogel was calculated to be 5.4~5.6 g·g^−1^. The experiment indicates that the surface-modified MSQ aerogels can selectively separate oil from oil/water mixtures with high efficiency due to their superhydrophobicity and superlipophilicity and could be applied as promising high efficiency oil adsorbents. MSQ aerogels with various chemical compositions and macroporous structures can be designed by adjusting the ratio of co-precursor, concentration of surface modifiers and solvent volume, and can be applied to meet various oil/water separation needs.

### 2.5. Fast Dye Adsorptionof New Flexible MSQ Aerogels

#### 2.5.1. Fast Dye Adsorption Facility

**Figure 11:**
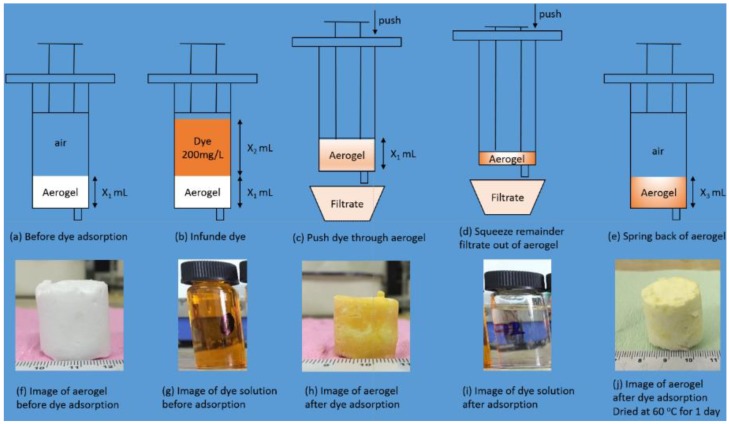
Sketch maps of fast dye adsorption facility.

With the development of industry, the treatment of organic dyes in waste water has been a serious environmental problem. Since the traditional powder adsorbents always suffer from the problem of low adsorption efficiency and adsorbent recovery, more and more researchers have focused on monolithic adsorbents for dyes [27,28,29,30,31], especially aerogel monoliths with high porosity and abundant groups. The great mechanical properties of MSQ aerogels determine that theyr can be easily assembled into monolithic columns, and their high porosity, superhydrophobicity and superlipophilicity offer possibilities for fast dye absorption. Figure 11 shows a sketch of a fast dye adsorption facility built by us. Firstly, a block of MSQ aerogel (6 mL) was put into a needle, which could be easily done because of its great flexibility. Secondly, 9 mL of dye solution (200 mg·L^−1^) was filled into the needle. Thirdly, the dye solution was pushed through the aerogel into a beaker. Then, the compressing aerogel was kept to squeeze out the filtrate into the beaker. After squeezing out all of filtrate, the core rod of the needle was lifted and the aerogel allowed to spring back. After springback, the volume of aerogel without surface modification was about 4.5 mL, which was smaller than that of aerogel with surface modification of up to 5 mL, resulting from its better elasticity and hydrophobicity. Finally, dye-absorbed aerogels were dried at 60 °C for 1 day and sprung back to the original size.

#### 2.5.2. Fast Dye Adsorption for Methyl Orange

**Figure 12:**
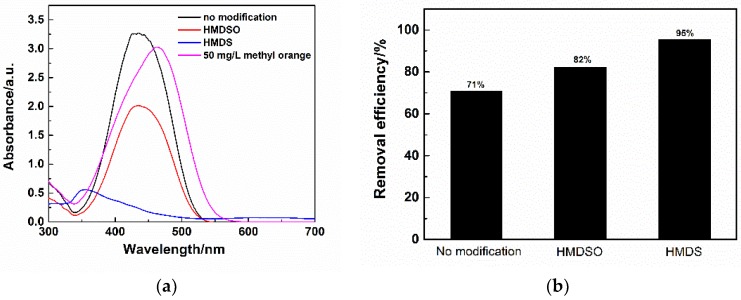
(**a**) UV visible absorption spectra of filtrates filtered by flexible MSQ aerogels (*X*_1_ = 6 mL) with different surface modifications and 50 mg·L^−1^ methyl orange standard solution; (**b**) Removal efficiency of flexible MSQ aerogels with different surface modifications.

The removal efficiency of surface modified flexible MSQ aerogels has been measured and compared. Figure 12a shows the UV visible absorption spectra of filtrates filtered by flexible MSQ aerogels with different surface modifications and 50 mg·L^−1^ methyl orange standard solution. Compared with the standard curve, the peaks (*λ*_max_) on the curves of filtrates filtered by flexible MSQ aerogels have varied degrees of blue shift. λ_max_ on the curve of the aerogel without surface modification or HMDSO modification at about 430 nm, attributed to the dissolution of residual cationic surfactant (CTAC) [32]. The *λ*_max_ on the curve of aerogel at about 350 nm is due to the dissolution of residual Gemini surfactants (HMDS) [33]. Concentration of methyl orange filtrates can be calculated by the following equation fitted by the absorbance of standard methyl orange solution (Equation (2)), in which the Abs is the highest absorbance and the *C* is the concentration of methyl orange.:

Abs = *C* × 0.054739 + 0.086714 (2)

**Figure 13:**
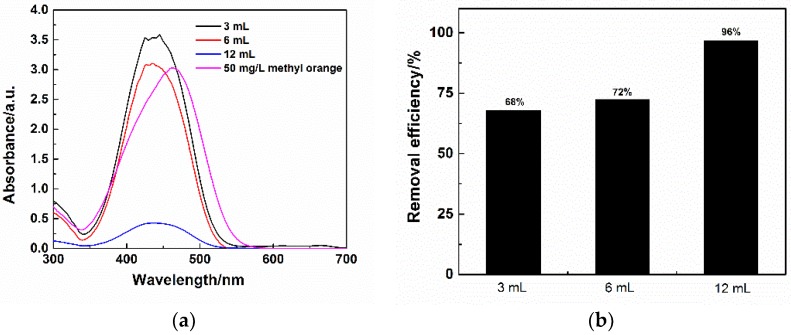
(**a**) UV visible absorption spectra of filtrates filtered by varied volumes of flexible MSQ aerogels without surface modification and 50 mg·L^−1^ methyl orange standard solution; (**b**) Removal efficiency of varied volumes’ flexible MSQ aerogels without surface modification.

According to the concentration of filtrates, the removal efficiency of MSQ aerogels is calculated and shown in Figure 12b. MSQ aerogel (6 mL) with surface modification of HMDS possesses the highest removal efficiency up to 95%. The influence of MSQ aerogels’ volume on removal efficiency for methyl orange also has been investigated. Figure 13a shows the UV visible absorption spectra of filtrates filtered by varied volumes of flexible MSQ aerogels without surface modification. Figure 13b shows the removal efficiency of varied volumes of flexible MSQ aerogels without surface modification. With the increase of MSQ aerogels’ volume, the removal efficiency gradually rises. When the volume of MSQ aerogel is 12 mL, the removal efficiency is up to 96%.

#### 2.5.3. Fast Dye Adsorption for Varied Dye Solutions

In view of the high removal efficiency of MSQ aerogels for methyl orange, fast dye absorption for other dye solutions was also investigated, including Congo Red, Rhodamine B and methylthionine chloride.

**Figure 14:**
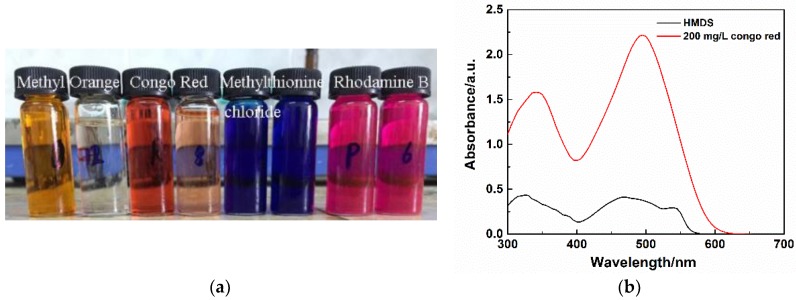
(**a**) Digital camera image of different dye solutions before and after absorption of flexible MSQ aerogel with HMDS surface modification; (**b**) UV visible absorption spectra of Congo Red filtrates filtered by flexible MSQ aerogels with HMDS surface modification.

Figure 14a shows digital camera images of dye solutions before and after absorption on flexible MSQ aerogel with HMDS surface modification. Obviously, there is no absorption for Rhodamine B and methylthionine chloride. Figure 14b shows the UV visible absorption spectra of Congo Red filtrates filtered by flexible MSQ aerogels with HMDS surface modification. The removal efficiency of MSQ aerogels surface-modified by HMDS is calculated to be about 80%, which proves that MSQ aerogels possesses selective adsorption for anionic dyes.

## 3. Experimental Section

### 3.1. Aerogel Synthesis

Methyltrimethoxysilane (MTMS, Aladdin, Shanghai, China, 98%), dimethyldimethoxysilane (DMDMS, Aladdin, 97%), hydrochloric acid (HCl, Aladdin), hexadecyltrimethylammonium chloride (CTAC, Aladdin, 97%), propylene oxide (PO, Sinopharm Chemical Reagent Co., Ltd., Shanghai, China, ≥99.5%), 2-propanol (IPA, Sinopharm Chemical Reagent Co., Ltd., ≥99.7%), hexamethyl- disiloxane (HMDSO, Aladdin, 99%), hexamethyldisilazane (HMDS, Aladdin, 98%) were used as received.

A typical flexible MSQ aerogel was prepared using 2 mL of co-precursors (MTMS and DMDMS), 4~8 mL of 0.0005 M HCl solution, 0.32 g of CTAC, and 1.5 mL of PO. Firstly, CTAC and HCl solution were mixed in a glass tube and then co-precursors were added with vigorous stirring at room temperature. After continuously stirring for 60 min, propylene oxide was added to the transparent solution under ambient conditions (25 °C). After stirring for 1 min, the resultant solution was allowed to gelate at 40 °C under closed conditions. The resultant gel was aged over 12 h at the same temperature and solvent exchanged in 2-propanol (IPA) three times. After solvent exchange, the alcohol gel was slowly dried at 40 °C for 1 day (at atmospheric pressure). The volume ratios of DMDMS and MTMS was set at 0, 0.6 and 1, and the volume of HCl solution was set at 4, 6 and 8 mL, respectively.

### 3.2. Surface Modification of Aerogels

Some of the wet gel samples which had been solvent exchanged by IPA were soaked in the following six kinds of surface modification solutions: (a) HMDSO; (b) HMDS; (c) HMDSO + HMDS (volume ratio is 1:1); (d) HMDSO + IPA (volume ratio is 0.4:10); (e) HMDS and IPA (volume ratio is 0.4:10); (f) HMDSO, HMDS and IPA (volume ratio is 0.2:0.2:10). After surface modification twice, the resultant gel samples were soaked in IPA once for 12 h, and then slowly dried at 40 °C for 1 day (atmospheric pressure) to get surface-modified aerogels.

### 3.3. Characterization

Microstructure of flexible MSQ aerogels was observed by scanning electron microscope (SEM: Su8010, Hitachi, Tokyo, Japan). Bulk density was calculated from the weight/volume ratio of the aerogel samples. Chemical compositions of flexible MSQ aerogels were measured by X-ray photoelectron spectroscopy (XPS: 250XI, ThermoFisher, Waltham, MA, USA). Elasticity (stress-strain curves) of flexible MSQ aerogels was characterized by universal material testing machine (Zwick/Roell Z020, Zwick, Ulm, Germany). Hydrophobicity (water contact angle) of flexible MSQ aerogels was performed by a video-based contact angle measuring device (OCA20, Dataphysics, Stuttgart, Germany). Acoustic absorptivity of flexible MSQ aerogels was measured by the standing wave tube method. Infrared (IR) transmittance spectra of flexible MSQ aerogels after dye absorbance was carried out by using the standard KBr-pellet technique with an infrared analyzer (FT-IR: Nicolet5700, ThermoFisher). Dye concentration was measured on an ultraviolet visible spectrophotometer (UV-Vis: UV6100, Mapada, Shanghai, China). Oil/water separation and fast dye adsorption were characterized by our self-designed method.

## 4. Conclusions

New flexible MSQ aerogel monoliths have been prepared by a sol–gel process followed by surface modification and ambient pressure drying. Appropriate volume ratios of MTMS and DMDMS co-precursors and solvent volume allow the formation of flexible MSQ aerogels. The MSQ aerogels after surface modification with HMDS possesses an elastic limit up to 70%, a water contact angle up to 157°, a density of 0.12 g·cm^−3^ and a porosity of 94%, and exhibit good performance in sound absorption, oil/water separation and selectively absorption of anionic dyes. The acoustic absorptivity of the surface-modified MSQ aerogel at both intermediate and high frequency is up to 80%. The oil absorption capacity of surface-modified MSQ aerogel is measured by a self-designed method and it is calculated to be 5.4~5.6 g·g^−1^. Meanwhile, the surface-modified MSQ aerogels are also found to have selective adsorption for anionic dyes with a removal efficiency of Methyl Orange up to 95%.

## Figures and Tables

**Table 1:** Atomic percentage of flexible MSQ aerogels without or with modifications calculated by XPS.

Sample	Surface Modification	C1s%	O1s%	Si2p%	Surface Composition (3~5 nm)
(1)	no	54.26	25.51	20.23	SiO_1.26_(CH_3_)_2.68_
(2)	HMDSO	51.82	27.48	20.7	SiO_1.35_(CH_3_)_2.50_
(3)	HMDS	55.21	25.57	19.22	SiO_1.33_(CH_3_)_2.87_

**Table 2:** Maximum strain, maximum stress and young modulus of flexible MSQ aerogels.

Sample	Surface Modification	F_max_ (MPa)	*dL*_max_ (%)	Young’s Modulus (KPa)
(1)	no	0.0325	60	0.215
(2)	HMDSO	0.0253	70	0.074
(3)	HMDS	0.0214	70	0.060
(4)	HMDSO + HMDS	0.0234	70	0.063
(5)	HMDSO + IPA	0.0248	60	0.163
(6)	HMDS + IPA	0.0433	70	0.141
(7)	HMDSO + HMDS+ IPA	0.0427	70	0.132
